# Selective Harvests of Brown Bears Associated With Increasing Skull Size and Older Age Structure

**DOI:** 10.1002/ece3.73468

**Published:** 2026-04-13

**Authors:** Jamshid Parchizadeh, Nathan J. Svoboda, Kenneth F. Kellner, Jon E. Swenson, Gary J. Roloff, Jerrold L. Belant

**Affiliations:** ^1^ Department of Fisheries and Wildlife Michigan State University East Lansing Michigan USA; ^2^ Alaska Department of Fish and Game Wildlife Division Kodiak Alaska USA; ^3^ Faculty of Environmental Sciences and Natural Resource Management Norwegian University of Life Sciences Ås Norway

**Keywords:** age, brown bear, guide, selective harvest, skull size, *Ursus arctos*

## Abstract

Legal harvests of wildlife occur throughout North America, where some big game hunters preferentially target individuals with larger body parts (e.g., horn, antler, body size), a practice referred to as selective harvest. Selective harvests have the potential to influence the phenotypic and age composition of harvested animals (e.g., body size, age structure), although inference about population‐level change from harvest data alone is inherently limited. Moreover, professional guides can play an important role in selective harvests by helping their clients more frequently harvest animals with larger body parts compared to hunters without guides. We used records of 6426 legally harvested brown bears ( *Ursus arctos* ) (4625 males; 1801 females) on the Kodiak Archipelago, Alaska, USA, from the 1987–2023 regulatory years to evaluate patterns consistent with two nonexclusive hypotheses regarding selective harvests. Based on the selective harvest hypothesis, we predicted that the selection of bears with larger skulls (i.e., older bears) by hunters would result in a decline in skull size and a younger age structure of harvested individuals through time. Based on the hunter origin effect hypothesis, we predicted that, because guides are generally more experienced and more familiar with hunt areas, guided hunters, on average, would harvest bears with larger skulls more often and in less time than unguided hunters. Contrary to expectations under selective harvests, we observed an increase of almost 2 years in age and 1.7 cm in skull size among harvested bears over the 37‐year period. Guided hunters, on average, harvested bears with larger skulls more frequently than unguided hunters but spent more days in the field before a successful harvest, compared to unguided hunters. Our results suggest temporal and hunter‐related patterns in characteristics of harvested bears and that current harvest regulations on the Kodiak Archipelago are associated with sustained availability of older, larger individuals in the harvested population.

## Graphical abstract

Figure 1 shows brown bear hunt areas on the Kodiak Archipelago, Alaska, USA.

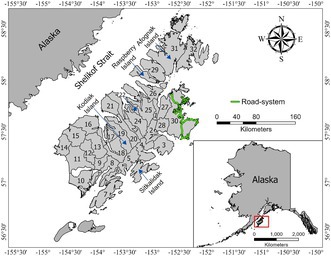

## 1 Introduction

Humans harvest wild animals for diverse reasons including recreation, food, and clothing (Allendorf and Hard 2009; Knell and Martínez‐Ruiz 2017). Harvest of wild animals by humans is frequently a major source of mortality for harvested species (Milner et al. 2011; Hill et al. 2019) and is often selective (Mysterud 2011). Selective harvests preferentially target individuals based on sex, age or age class, or body size and associated morphological traits (Fenberg and Roy 2008; Badenhorst 2015). In mammals, hunters often seek large‐bodied individuals or those with conspicuous body parts such as horns, antlers, or tusks (Festa‐Bianchet 2003; Knell and Martínez‐Ruiz 2017). Empirical examples include preferential harvest of bighorn sheep ( *Ovis canadensis* ) with larger horns (Coltman et al. 2003), elephants ( *Loxodonta cyclotis* ) with larger tusks (Dobson and Poole 1998), and larger‐bodied leopards ( *Panthera pardus* ; Bailey 2005).

Selective harvests may alter the phenotypic composition and age structure of harvested animals (Fenberg and Roy 2008; Allendorf and Hard 2009) where inference about underlying population‐level impacts remains challenging (Coltman et al. 2003, Milner et al. 2007, McLellan et al. 2017). The selective harvest hypothesis predicts that selective harvests of individuals with larger body parts favor survival of conspecifics with smaller body parts (younger individuals are generally smaller in size; Fenberg and Roy 2008, LaSharr et al. 2019), which in turn can lead to declines in body size or shifts toward younger age structures in harvested individuals over time (Hsieh et al. 2006; Bischof et al. 2008; Monteith et al. 2013; Barnett et al. 2017). This phenomenon has been observed in harvested marine and terrestrial animal populations (Coltman et al. 2003; Fenberg and Roy 2008; Schindler et al. 2017).

The hunter origin effect hypothesis posits that nonresident hunters harvest individual animals with larger body parts more frequently than resident hunters (Rivrud et al. 2013; Douhard et al. 2016). Nonresident hunters in some North American jurisdictions must be accompanied by registered guides while hunting big game (Douhard et al. 2016). Guides are typically experienced hunters (Albert et al. 2001) and proficient in locating animals with larger body parts in their hunt areas, which can facilitate guided nonresident hunters (hereafter guided hunters) harvesting individuals with larger body parts more frequently and in less time than unguided resident hunters (hereafter unguided hunters) (Reiling et al. 1991; Schmidt et al. 2007). Furthermore, guided hunters often harvest big game in part to acquire individuals considered a trophy (i.e., with large body parts) (Albert et al. 2001; Backman et al. 2001), whereas unguided hunters more frequently harvest big game primarily for reasons other than obtaining a trophy (Faunce et al. 1979; Backman et al. 2001; Aastrup et al. 2021). Consequently, guided hunters in British Columbia, Canada, harvested mountain goats ( *Oreamnos americanus* ) with larger horns more often than unguided hunters (Rice et al. 2022). Moreover, unguided hunters in Maine, USA spent an average of 9 days to harvest American black bears ( *Ursus americanus* ) and 12% were successful, whereas guided hunters spent an average of 5.5 days and 35% were successful (ElHamzaoui et al. 1994).

Brown bears ( *U. arctos* ) are harvested throughout Alaska, USA, where larger‐bodied individuals are often selected by hunters (Albert et al. 2001; Brockman et al. 2020). Our objective was to evaluate support for the nonexclusive selective harvest and hunter origin effect hypotheses using brown bear harvests on the Kodiak Archipelago, Alaska, which are managed under conservative harvest regulations (Van Daele and Barnes 2010). We predicted that, because selective harvests of larger‐bodied (older) brown bear individuals occur frequently by trophy hunters (Van Daele and Barnes 2010), we would observe a decline in body size as represented by skull measurements across years, as a positive correlation exists between brown bear skull size and body size (Rivrud et al. 2019; Todorov et al. 2022), or a younger age structure for harvested individuals across years. Alternatively, if selective harvests were at low enough levels, a decline in body size as indexed by skull size or younger age structure would not occur. We also predicted that, due to local knowledge of professional guides and their enhanced skills and experience, guided hunters on average would harvest bears with larger skulls more frequently than unguided hunters, and that guided hunters on average would spend fewer days in the field to harvest brown bears compared to unguided hunters.

## 2 Materials and Methods

### 2.1 Study Area

**FIGURE 1:**
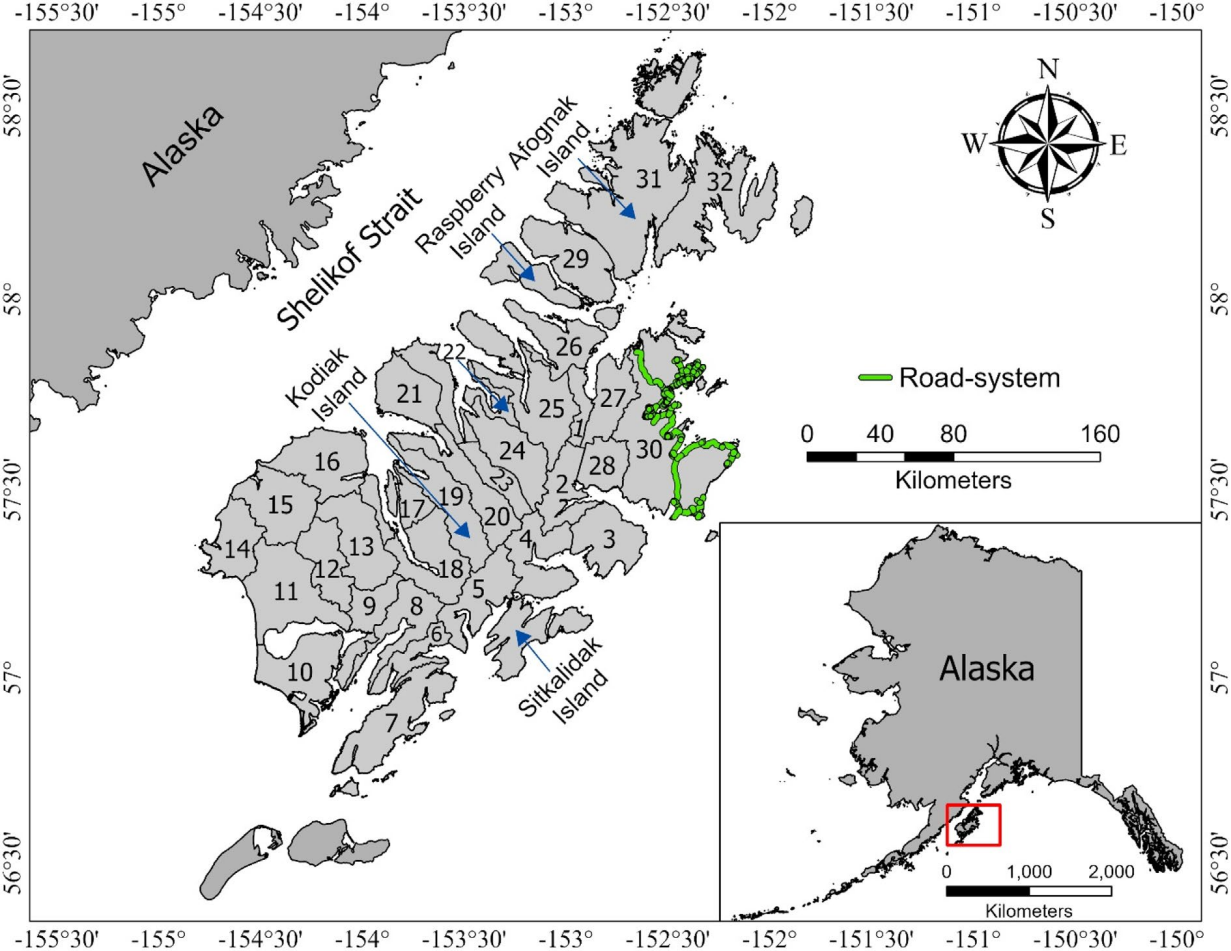
Brown bear ( *Ursus arctos* ) hunt areas, Kodiak Archipelago, Alaska, USA.

The Kodiak Archipelago is in the Gulf of Alaska, Alaska, USA (56°25′–58°40′ N, 152°00′–154°50′ W) and comprises 12,950 km^2^ (Van Daele and Barnes 2010; Figure 1). The main islands of the archipelago include Kodiak (8975 km^2^), Afognak (1809 km^2^), Sitkalidak (300 km^2^), and Raspberry (197 km^2^; Figure 1). There are about 77 km of roads on northeastern Kodiak Island (Van Daele and Barnes 2010). A human population of 12,193 individuals live along this road system (i.e., hunt area 30) and 685 people live in one of 6 remote villages (i.e., hunt areas 1–29, 31, and 32) (United States Census Bureau 2023).

Elevations are 0–1362 m above sea level (Cobb et al. 2012). The climate is subarctic maritime with long, wet winters and cool, wet summers; average annual low and high temperatures are 2.1°C and 8.0°C, respectively (Cobb et al. 2012; Schooler et al. 2022). Average annual rainfall and snowfall are 198 and 189 cm, respectively (United States Fish and Wildlife Service 2018). Sitka spruce (*Picea stichensis*) is a common tree species on Afognak and parts of northern Kodiak Island, whereas Kenai birch ( *Betula kenaica* ), Sitka alder (*Alnus crispa sinuata*), and willows (*Salix* spp.) are more common in other areas (Fleming and Spencer 2007). Salmonberry ( *Rubus spectabilis* ), blueberry ( *Vaccinium ovalifolium* ), and devil's club ( *Oplopanax horridus* ) are dominant understory species (Finnegan et al. 2021). Introduced terrestrial mammals include Sitka black‐tailed deer ( *Odocoileus hemionus* ), Roosevelt elk ( *Cervus canadensis* ), caribou ( *Rangifer tarandus* ), mountain goats, and American bison ( *Bison bison* ). Native small‐ and medium‐sized terrestrial mammals include red fox ( *Vulpes vulpes* ), North American river otter ( *Lontra canadensis* ), ermine ( *Mustela erminea* ), tundra vole (*Alexandromys oeconomus*), and the little brown bat ( *Myotis lucifugus* ). The brown bear is the only native large terrestrial mammal on the archipelago, with an estimated abundance of 3500 individuals (Van Daele 2007; Van Daele and Barnes 2010), representing 200–221 independent bears/1000 km^2^ (Van Daele et al. 2012).

Brown bears are legally harvested throughout the Kodiak Archipelago (Schooler et al. 2022). Each regulatory year (July 1–June 30; Alaska Department of Fish and Game 2023), the Alaska Department of Fish and Game (ADF&G) issues registration (i.e., open entry) permits to harvest bears in hunt area 30, whereas limited drawing (i.e., random lottery) permits are awarded to harvest bears in hunt areas 1–29, 31, and 32 (Figure 1). The number of permits issued is determined using population size estimates and harvest rates from the previous year (Van Daele and Barnes 2010; Svoboda and Crye 2023). Alaska state law requires nonresident hunters to hunt with registered guides (Van Daele and Barnes 2010). All hunters are limited to harvesting one bear every four regulatory years; harvest of adult females with young and their dependent young is prohibited (Schooler et al. 2022). Hunters in the drawing hunt are limited to a 15‐day consecutive hunt period (Alaska Department of Fish and Game 2023). Harvests occur during fall (October 25–November 30) and spring (April 1–May 15) each regulatory year (Alaska Department of Fish and Game 2023).

### 2.2 Harvest Data

Hunters are required to present the hide and skull of harvested bears to ADF&G personnel for inspection and Convention on the International Trade of Endangered Species sealing (Van Daele and Barnes 2010) before the hide or skull is transported from the archipelago. At the time of sealing, ADF&G personnel collect data on each harvested bear. A vestigial upper premolar is extracted to estimate bear age using cementum annuli (Calvert and Ramsay 1998). Maximum skull length (i.e., straight‐line distance from the upper incisors to the occipital condyle) and zygomatic width (i.e., maximum straight‐line distance at the zygomatic arches) (Hilderbrand et al. 2018; Brockman et al. 2020) are recorded and summed to portray total skull size. Additionally, bear sex (female or male), hunting season (fall or spring), regulatory year, hunter residency status (resident or nonresident), number of days in the field to harvest (i.e., number of days hunted), and hunt area are recorded (Alaska Department of Fish and Game 2023). We analyzed records of harvested brown bears sealed by ADF&G personnel from the 1987–2023 regulatory years.

### 2.3 Analyses

Brown bear skull size and age are both indices of body size (Rivrud et al. 2019) and thus are positively correlated (e.g., Bartareau et al. 2011; Svoboda and Crye 2023). Nevertheless, we examined both metrics by fitting separate linear mixed effects models, one with skull size as the response and one with age as the response. The two models had the same covariate and random effect structure. Employment of professional guides is expensive in Alaska (Schmidt et al. 2007), leading to a small portion of resident hunters (about 1%) hiring guides (Albert et al. 2001). As nonresident hunters are required to hire guides (Van Daele and Barnes 2010), we categorized nonresident hunters as guided and assumed resident hunters were unguided. We used regulatory year, hunter guide status, bear sex, and hunting season as fixed effects, and included hunt area as a random effect using the function *lmer* in the lme4 package (Bates and Sarkar 2007) in program R version 4.1.1 (R Core Team 2023). We plotted predicted total skull size and age as a function of regulatory year for each sex using the function *plot_model* in the sjPlot package (Lüdecke et al. 2024). We plotted predicted mean values and 95% confidence intervals for total skull size as functions of guide status, and season, using the function *plot_model* in the sjPlot package (Lüdecke et al. 2024). We used Wald tests to determine if covariates had a statistically significant effect on the response (*p* < 0.05). We reported all means within one standard deviation (±).

To test for differences in hunt duration as a function of guided status, we fit a linear mixed effect model with number of days hunted as a continuous response variable. Covariates in this model included hunter guide status, bear sex, and hunting season as fixed effects. Hunt area and regulatory year were random effects in the model. We plotted predicted mean values and 95% confidence intervals for the number of days hunted as functions of guide status, sex, and season.

Because variation in skull size and age at harvest during the study period could be related to the number of bears harvested annually, we examined potential changes in harvest numbers across regulatory years (i.e., to test whether skull and ages represented similar levels of sampling over time) using a linear regression model. The number of harvested bears per regulatory year was the response variable, and regulatory year was the covariate. We fit this model using the function *lm* in the stats package (R Core Team 2023).

Male brown bears, particularly adult males, are typically more vulnerable to harvest during spring hunting seasons (Glenn and Miller 1980; Miller 1990), which might result in age and skull size of harvested bears being greater on average in spring than in fall. Because this could be linked to earlier den emergence of males compared to females (Schoen et al. 1987; Mangipane et al. 2018), we compared timing of harvests (i.e., earlier or later in the season) between male and female bears in fall and spring seasons. We converted calendar dates to sequential day numbers. For the fall season, we assigned Day 1 to October 25 and Day 37 to November 30. For the spring season, we assigned Day 1 to April 1 and Day 45 to May 15. We then fit separate linear mixed models for each season using sequential date numbers (continuous response variables) as a function of bear sex and included hunt area and regulatory year as random effects in the model.

## 3 Results

**FIGURE 2:**
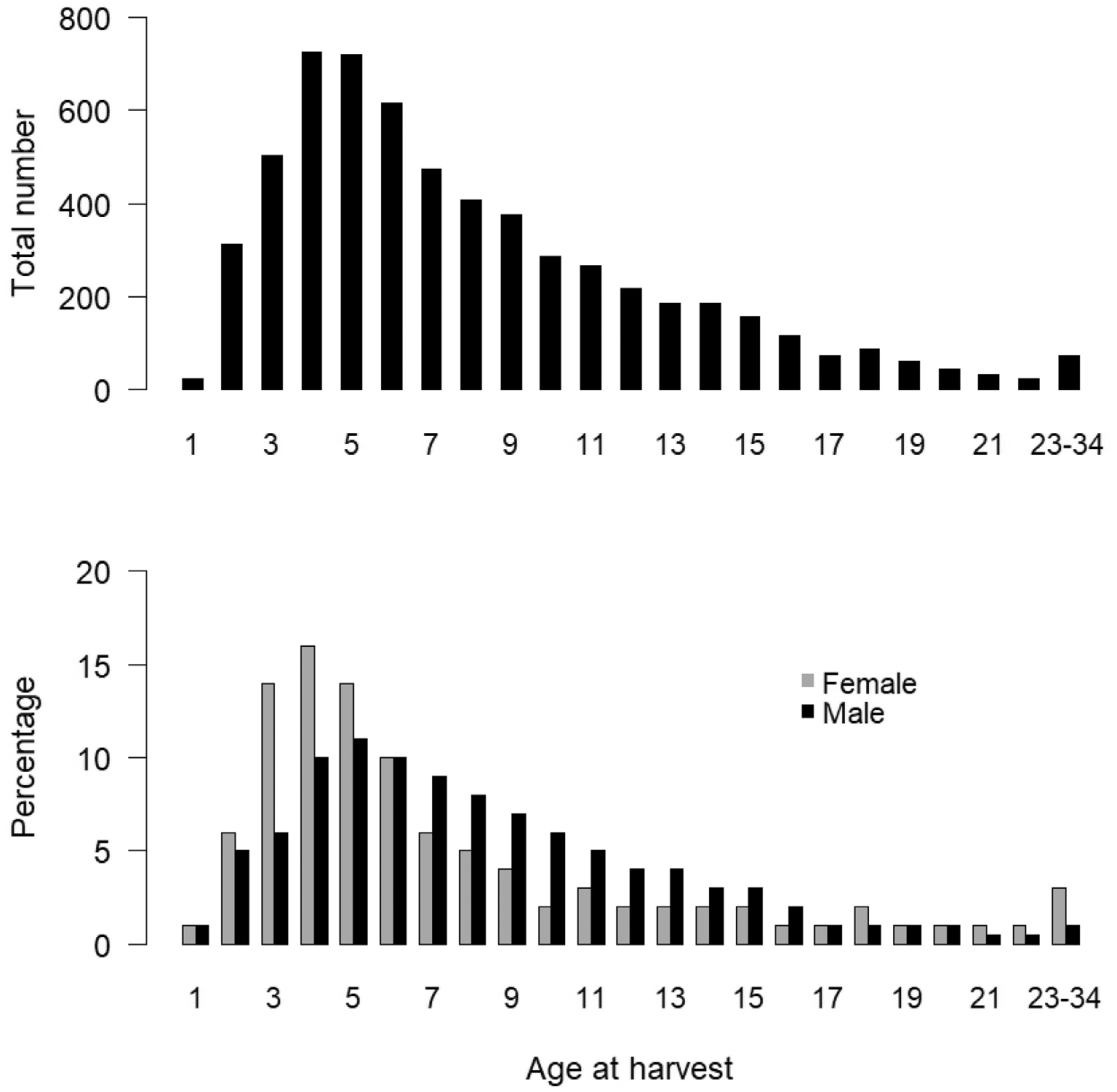
Age of brown bears ( *Ursus arctos* ) at harvest by total number of individuals (top panel), and distribution of ages for females and males by percentage (bottom panel), Kodiak Archipelago, Alaska, USA, 1987–2023 regulatory years (July 1–June 30).

Our dataset included records of 6426 legally harvested brown bears (Table 1). Ages of harvested bears were 1–34 years old (Figure 2). Number of harvested bears among regulatory years ranged from 68 to 251 with a mean of 174 (SD = 33) individuals (Table 2). For fall hunting seasons, mean annual percentages of harvested males and females were 67% (SD = 7%) and 33% (SD = 8%), respectively. For spring hunting seasons, the mean annual percentage of harvested males was 75% (SD = 14%) and the mean annual percentage of females was 25% (SD = 7%).

**TABLE 1:** Number, percentage, and sex ratio for harvested brown bears ( *Ursus arctos* ) during fall (October 25–November 30) and spring (April 1–May 15) hunting seasons, Kodiak Archipelago, Alaska, USA, 1987–2023 regulatory years (July 1–June 30).

Season	Number of males (%)	Number of females (%)	Total number (%)	Male: female ratio
Fall	1555 (66)	788 (34)	2343 (36)	2.0:1.0
Spring	3070 (75)	1013 (25)	4083 (64)	3.0:1.0
Total	4625 (72)	1801 (28)	6426 (100)	2.6:1.0

**TABLE 2:** Mean number and standard deviation (SD) of harvested brown bears ( *Ursus arctos* ) and hunters during fall (October 25–November 30) and spring (April 1–May 15) hunting seasons across regulatory years (July 1–June 30), Kodiak Archipelago, Alaska, USA, 1987–2023.

Season	Male bears (SD)	Female bears (SD)	All bears (SD)	Successful hunters (SD)	Unsuccessful hunters (SD)	All hunters (SD)
Fall	42 (11)	21 (7)	63 (15)	63 (15)	119 (41)	182 (49)
Spring	83 (23)	28 (9)	111 (28)	111 (28)	130 (56)	241 (71)
Total	125 (28)	49 (12)	174 (33)	174 (33)	249 (75)	423 (95)

**FIGURE 3:**
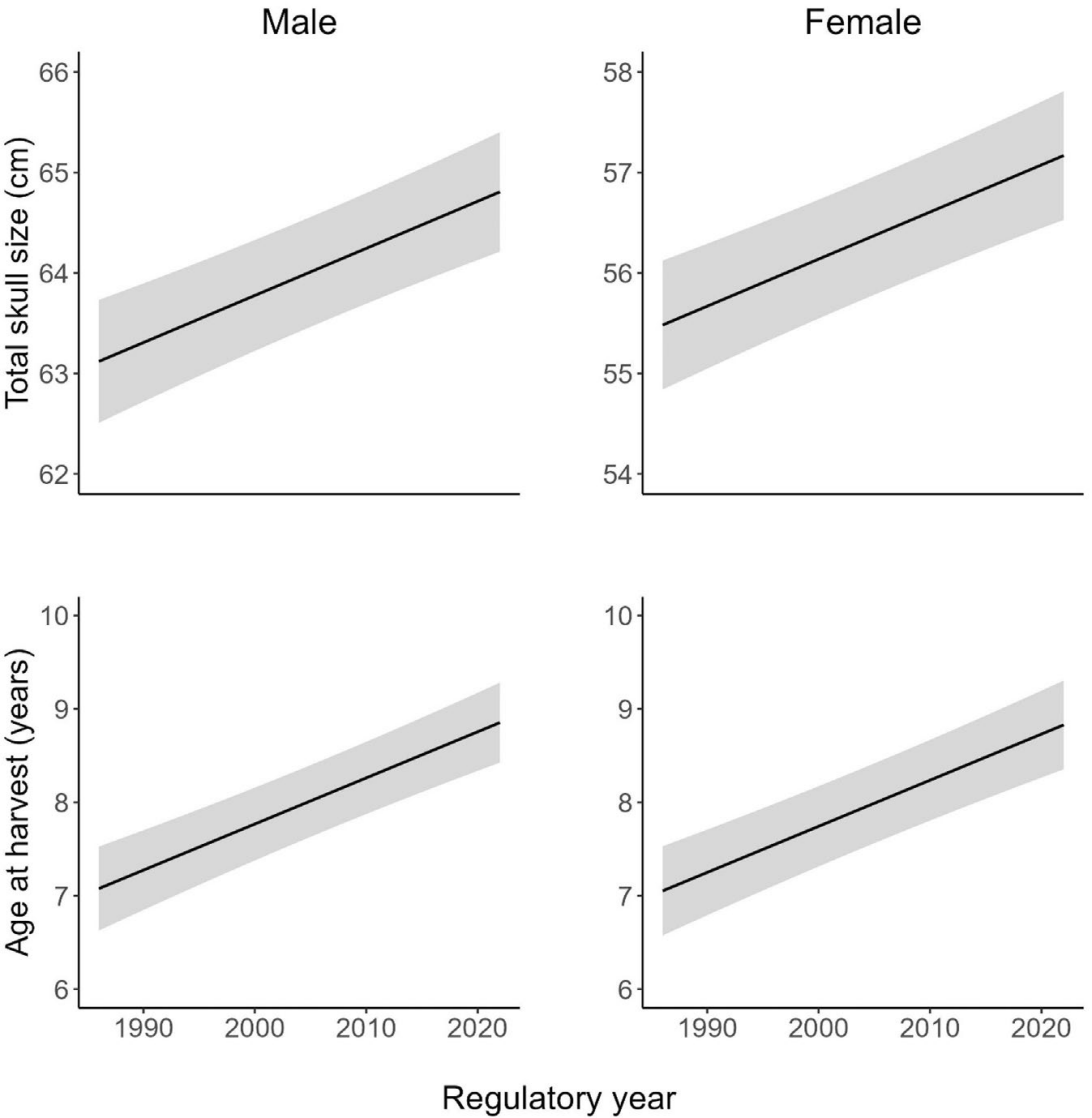
Model‐predicted responses for total skull size (maximum skull length plus zygomatic width) of harvested male brown bears ( *Ursus arctos* ) and their age at harvest (left panels), and those of harvested females (right panels) by regulatory year (July 1–June 30), Kodiak Archipelago, Alaska, USA, 1987–2023 regulatory years. Shaded areas represent 95% confidence intervals around the predicted value.

**FIGURE 4:**
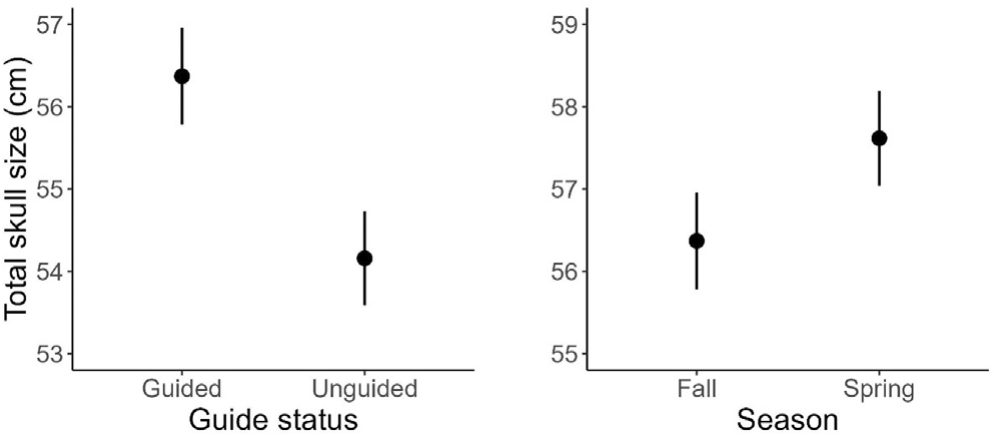
Predicted mean values and 95% confidence intervals for total skull size (maximum skull length plus zygomatic width) of harvested brown bears ( *Ursus arctos* ) in relation to hunter guide status, and hunting season (fall [October 25–November 30], spring [April 1–May 15]), Kodiak Archipelago, Alaska, USA, 1987–2023 regulatory years (July 1–June 30).

We found a significant effect of regulatory year, guide status, sex, and season on total skull size (Table 3). We also found a significant effect of regulatory year, guide status, and season on age at harvest. Estimated mean skull size increased from 63.12 to 64.81 cm for harvested males, and from 55.48 to 57.17 cm for harvested females (Figure 3). For both sexes, estimated mean age of harvested individuals increased from 7 years old to almost 9 years old (Figure 3). Harvested bears by guided hunters were 2.21 cm larger in total skull size (Table 3, Figure 4) and 1.66 years of age older than those by unguided hunters. Harvested bears in spring had total skull size 1.25 cm larger and were 1.07 years older than bears harvested in fall. Harvested male bears were 7.64 cm larger than females overall, but age at harvest was similar (mean for males and females = 8 years).

**TABLE 3:** Estimated parameters from two linear mixed models of brown bear ( *Ursus arctos* ) total skull size (cm) (maximum skull length plus zygomatic width) and age at harvest (years) as a function of regulatory year (July 1–June 30), hunter guide status (guided or unguided), bear sex (female or male), and hunting season (fall [October 25–November 30] or spring [April 1–May 15]), with hunt area as random effect, Kodiak Archipelago, Alaska, USA, 1987–2023 regulatory years. Reference levels included guide status (unguided), sex (female), and season (fall).

Response	Parameter	Estimate	95% CI	SE	*Df*	*p*
Total skull size	Intercept	−34.90	−61.71, −8.02	13.70	6177	0.011
	Regulatory year	0.04	0.03, 0.06	0.01	6174	< 0.001
	Guide status (guided)	2.21	1.93, 2.49	0.14	6184	< 0.001
	Sex (male)	7.64	7.33, 7.95	0.16	6169	< 0.001
	Season (spring)	1.25	0.96, 1.53	0.15	6181	< 0.001
Age at harvest	Intercept	−92.61	−116.15, −68.97	12.03	6114	< 0.001
	Regulatory year	0.05	0.04, 0.06	0.01	6113	< 0.001
	Guide status (guided)	1.66	1.42, 1.90	0.12	6118	< 0.001
	Sex (male)	0.02	−0.24, 0.29	0.14	6106	0.856
	Season (spring)	1.07	0.82, 1.32	0.13	6118	< 0.001

**FIGURE 5:**
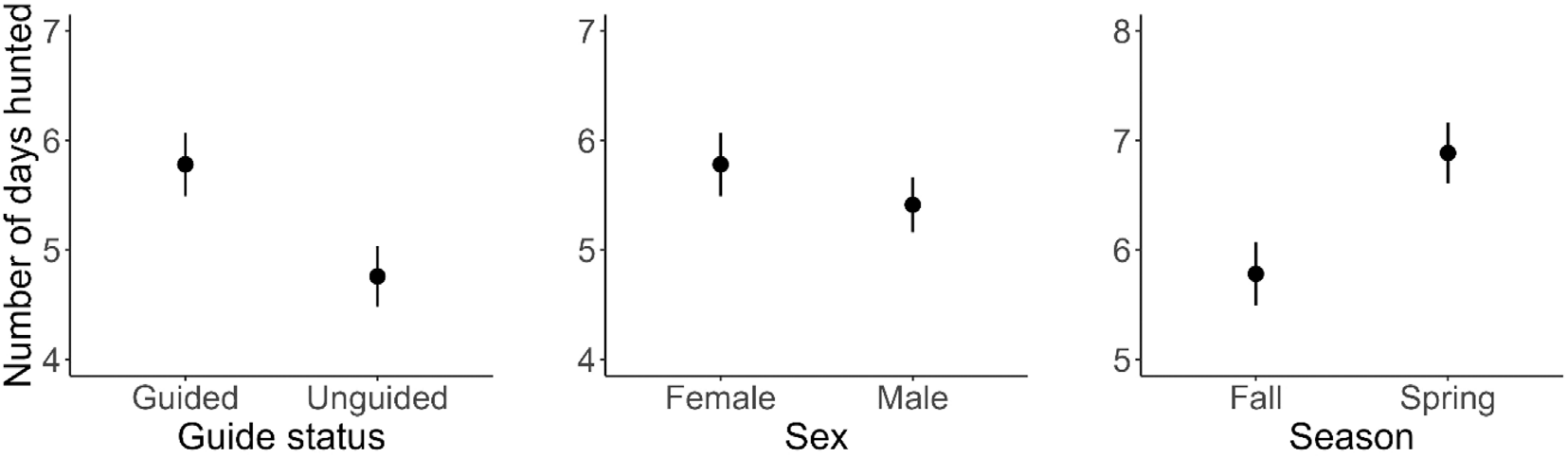
Predicted mean values and 95% confidence intervals for number of days brown bear ( *Ursus arctos* ) hunters spent to harvest a bear in relation to hunter guide status, bear sex, and hunting season (fall [October 25–November 30], spring [April 1–May 15]), Kodiak Archipelago, Alaska, USA, 1987–2023 regulatory years (July 1–June 30).

We found a significant effect of guide status, sex, and season on number of days hunted (Table 4). Successful guided hunters spent 1.02 days longer in the field compared to successful unguided hunters (Table 4, Figure 5). Male bears were harvested in less time (i.e., 0.37 fewer days) than females. Successful hunters spent 1.10 days longer hunting in spring than in fall.

**TABLE 4:** Estimated parameters from a linear mixed model of number of days brown bear ( *Ursus arctos* ) hunters spent to harvest a bear as a function of hunter guide status (guided or unguided), bear sex (female or male), and hunting season (fall [October 25–November 30] or spring [April 1–May 15]), with hunt area and regulatory year (July 1–June 30) as random effects, Kodiak Archipelago, Alaska, USA, 1987–2023 regulatory years. Reference levels included guide status (unguided), sex (female), and season (fall).

Parameter	Estimate	95% CI	SE	*Df*	*p*
Intercept	4.76	4.48, 5.03	0.14	148	< 0.001
Guide status (guided)	1.02	0.83, 1.21	0.10	6326	< 0.001
Sex (male)	−0.37	−0.58, −0.16	0.11	6343	< 0.001
Season (spring)	1.10	0.91, 1.30	0.10	6171	< 0.001

We found no change in the annual number of harvested bears during the 1987–2023 regulatory years (β = 0.50, 95% CI = −0.54–1.55, S.E. = 0.51, *p* = 0.335). Timing of harvests was similar for males and females during fall (mean date for both sexes = October 31), whereas males were harvested on average 1 day earlier than females during spring (mean date for males = May 1, mean date for females = May 2; Table 5).

**TABLE 5:** Estimated parameters from two linear mixed models for timing of harvests (day) during fall (October 25–November 30) and spring (April 1–May 15) hunting seasons as a function of brown bear ( *Ursus arctos* ) sex (female or male), with hunt area and regulatory year (July 1–June 30) as random effects, Kodiak Archipelago, Alaska, USA, 1987–2023. For the fall hunting season, Day 1 was assigned to October 25 and Day 37 was assigned to November 30. For the spring hunting season, Day 1 was assigned to April 1 and Day 45 was assigned to May 15. Reference level included sex (female).

Response	Parameter	Estimate	95% CI	SE	*Df*	*p*
Day in fall	Intercept	8.87	8.05, 9.69	0.41	67	< 0.001
	Sex (male)	−0.13	−0.75, 0.49	0.32	2322	0.683
Day in spring	Intercept	31.98	31.12, 32.85	0.44	72	< 0.001
	Sex (male)	−0.81	−1.38, −0.24	0.29	4041	0.006

## 4 Discussion

In contrast to expectations under the selective harvest hypothesis, mean skull size and age of harvested brown bears increased during the study period. Our findings contrast with patterns of selective harvests observed in other large mammal species (e.g., American black bear; Kolenosky 1983, white‐tailed deer [ *O. virginianus* ]; Jenks et al. 2002, bighorn sheep; Schindler et al. 2017). Our findings supported our alternative hypothesis suggesting that selective harvests were at low enough levels that a decline in body size as indexed by skull size or younger age structure did not occur. Most wildlife management agencies in North America including ADF&G use a conservative approach to set harvest regulations for brown bears (Miller 1990; Van Daele and Barnes 2010; McLellan et al. 2017). These regulations are often set based on maximum human‐caused mortality rate of 4.0%–6.5% of the brown bear population (Harris 1984, 1986; McLellan et al. 2017). An estimated annual harvest rate of 5% (i.e., 174 bears per regulatory year of the estimated total population of 3500 individuals for the Kodiak Archipelago [Van Daele 2007, Van Daele and Barnes 2010]) aligns with suggested harvest rates for brown bears. This in turn might have resulted in selective removal of few larger (older) bears by hunters each regulatory year on the archipelago (Van Daele and Barnes 2010), allowing remaining individuals to increase in body size and age. Van Daele (2007) concluded that the number of Kodiak brown bears harvested remained relatively consistent where males dominated the harvest (because male brown bears are larger than females, trophy hunters target males more often; Dahle and Swenson 2003). Van Daele (2007) further documented an increase in the number and the percentage of the harvest that consisted of trophy‐sized (total skull size > 71 cm) males. Observed increases in bears with large skulls from our study and Van Daele (2007) could reflect changes in hunter behavior, improved ability to identify larger bears, increased selectivity, or regulatory and educational efforts that encourage restraint and accurate identification of legal animals (Alaska Department of Fish and Game 2023). For instance, ADF&G educates all hunters before hunting by providing them booklets to identify brown bears by sex and age class (subadult and adult) (Alaska Department of Fish and Game 2023). Additionally, ADF&G personnel show hunters a taxidermy mount of a large bear to demonstrate what can be considered a large brown bear on the Kodiak Archipelago (Alaska Department of Fish and Game 2023). These factors might have contributed to the 3% increase in total skull size of harvested males and females during our study.

Consistent with our prediction, we found that on average, guided hunters harvested bears with larger skulls (and presumably larger bodies) and older bears more frequently than unguided hunters. This was likely due to local knowledge of guides and their ability to locate larger individuals (Schmidt et al. 2007; Leorna et al. 2020). A mandatory requirement for nonresident hunters of brown bears in Alaska is to hire a licensed guide (Albert et al. 2001; Rice et al. 2022). Guides commonly guide in the same area across years and become proficient in locating larger individuals, resulting in guided hunters harvesting these individuals more often compared to unguided hunters (Schmidt et al. 2007; Barnes and Novelli 2008; Leorna et al. 2020). For instance, guided hunters harvested larger‐bodied American black bears (Hasbrouck 2020) and mountain goats with larger horns (Rice et al. 2022) than unguided hunters.

We found that age and skull size of harvested bears were greater on average in spring than in fall. Male bears also were harvested slightly earlier than females during spring. Male brown bears (particularly adults) typically emerge from dens earlier than females in spring (e.g., Schoen et al. 1987; Manchi and Swenson 2005; Mangipane et al. 2018). Adult male bears on the Kodiak Archipelago have a mean den emergence date (April 14) 17 days earlier than solitary adult females (April 30; Parchizadeh et al. 2026). Furthermore, Van Daele et al. (1990) concluded that 22% of Kodiak male brown bears in their study did not den. This suggests that hunters would encounter adult males more frequently than females that are legal to harvest during spring, particularly early in the season, leading to greater vulnerability of this class (Miller 1990). For instance, adult male brown bears were harvested more often during spring hunting seasons (Glenn and Miller 1980), and 74% of brown and 75% of American black bears in Alaska harvested during 1984–1988 spring seasons were male (Miller 1990). Similarly, 75% of harvested bears in our study during 1987–2023 spring hunting seasons were male.

In contrast to our prediction, successful guided hunters on average spent more days to harvest a bear compared to successful unguided hunters, probably an outcome of higher selectivity by guided hunters. Unguided hunters are more likely to harvest any legal bear they encountered, whereas guided hunters often would not harvest smaller‐bodied bears they encountered and wait for a larger‐bodied individual (Miller et al. 1998; Auger 2006; Eliason 2008). Furthermore, guided hunters on the Kodiak Archipelago are required to pay for a 10‐day (on average) hunt in advance, which likely motivates them to wait longer to encounter and harvest larger bears. This could explain the greater number of days for guided hunters to harvest a bear compared to unguided hunters and underscores the importance of considering hunter behavior and goals when interpreting harvest‐derived data.

Our results revealed that on average, male bears were harvested sooner (i.e., in fewer days) compared to females. Males (particularly adults) have larger home ranges than females (e.g., Barnes 1990; McLoughlin et al. 2003; Van Daele 2007), leading to increased movement (i.e., travel greater distances) to meet bioenergetic and mating demands (McLoughlin et al. 1999; Ordiz et al. 2014). Differences in home range sizes and movement rates of males and females might have contributed to increased likelihood of encountering hunters for males and greater vulnerability to harvests for this class (Bunnell and Tait 1985; Miller et al. 2003). Alternatively, though harvest of large males is the goal of most brown bear hunters on the Kodiak Archipelago (Van Daele and Barnes 2010), it is possible that hunters who were unable to locate and harvest large males would consider harvesting a smaller female. This in turn could result in females being harvested later than males in the season.

Successful hunters on average spent more days in the field to harvest bears during spring than in fall. Spring weather in the Kodiak Archipelago is highly variable, and snow, rain, or melting ice can limit hunter movements, and fog can limit visibility (Smith 1979). Thus, spring weather can cause more difficult hunting conditions, resulting in more days required to harvest bears. Alternatively, weather conditions and food availability in spring are linked to den emergence in bears (Pigeon et al. 2016). Adverse weather conditions, longer duration of snow cover, and limited food availability could increase denning duration and delay den emergence (Fowler et al. 2019), whereas mild weather can have the opposite effect (Pigeon et al. 2016). For instance, when spring snowmelt occurs and when brown bears emerge from their dens in Alaska were positively correlated (Schoen et al. 1987). Growing season (i.e., the longest continuous period of nonfreezing temperatures in the year) usually starts in late April on the Kodiak Archipelago (Fitzhugh 2003), where subadult and adult brown bears (male and female) have a mean emergence date of April 21 (Parchizadeh et al. 2026). This suggests that bears emerged from dens approximately at the onset of the growing season, which might have contributed to the longer days for hunters to locate and harvest bears in spring than in fall.

## 5 Management Implications

Long‐term harvest records from the Kodiak Archipelago indicate that harvested brown bears have on average become older and larger over time, and that guided hunters consistently harvested larger individuals than unguided hunters. These patterns suggest that current harvest regulations are associated with continued availability of older, larger bears for trophy hunters. Notably, because our analyses are based exclusively on harvested individuals, they do not provide direct evidence of population‐level phenotypic stability, demographic resilience, or evolutionary responses. Continued monitoring that integrates harvest data with independent population estimates will be essential for evaluating long‐term sustainability and informing adaptive management of Kodiak brown bears. We also recommend further research to clarify the mechanisms underlying the observed patterns in our study.

## Author Contributions

**Jamshid Parchizadeh:** conceptualization (equal), formal analysis (equal), methodology (equal), software (equal), supervision (equal), visualization (equal), writing – original draft (equal), writing – review and editing (equal). **Nathan J. Svoboda:** conceptualization (equal), writing – review and editing (equal). **Kenneth F. Kellner:** conceptualization (equal), methodology (equal), writing – review and editing (equal). **Jon E. Swenson:** conceptualization (equal), writing – review and editing (equal). **Gary J. Roloff:** conceptualization (equal), writing – review and editing (equal). **Jerrold L. Belant:** conceptualization (equal), funding acquisition (equal), methodology (equal), supervision (equal), writing – review and editing (equal).

## Funding

This work was supported by the Alaska Department of Fish and Game, Pittman‐Robertson funds, and Boone and Crockett Program in Wildlife Conservation at Michigan State University.

## Conflicts of Interest

The authors declare no conflicts of interest.

## Data Availability

Data were provided by the State of Alaska and can be obtained through a formal data sharing agreement with the Alaska Department of Fish and Game.
